# Supplementary material for: Maternal Obesity and Offspring Long-Term Infectious Morbidity
Source: J Clin Med. 2019 Sep 14;8(9):1466. doi: 10.3390/jcm8091466 (PMC6780342; doi:10.3390/jcm8091466)
Supplement: Supplementary file 1 [file jcm-08-01466-s001.pdf]

Supplementary Table – ICD-9 Codes of infectious morbidity

Infectious Group	Diag. code	Diagnosis description
**Urinary tract infections**	5901	Acute pyelonephritis
	5950	Acute cystitis
	5959	Cystitis, unspecified
	5970	Urethral abscess
	5990	Urinary tract infection, site not specified
	59010	Ac.pyelonephritis without lesion of renal medullary necrosis
	59080	Pyelonephritis, unspecified
	59581	Cystitis cystica
	59589	Other specified types of cystitis
	59780	Urethritis, unspecified
	59789	Other urethritis
	V1302	Personal history of urinary (tract) infection
**Gastrointestinal infection**	008	Intestinal infections due to other organisms
	0030	Salmonella gastroenteritis
	0039	Salmonella infection, unspecified
	0040	Shigella dysenteriae
	0041	Shigella flexneri
	0042	Shigella boydii
	0043	Shigella sonnei
	0048	Other specified shigella infections
	0049	Shigellosis, unspecified
	0051	Botulism
	0059	Food poisoning, unspecified
	0068	Amebic infection of other sites
	0069	Amebiasis, unspecified
	0070	Balantidiasis
	0071	Giardiasis
	0078	Other specified protozoal intestinal diseases
	0079	Unspecified protozoal intestinal disease
	0084	Intestinal infection due to other specified bacteria
	0085	Bacterial enteritis, unspecified
	0088	Intestinal infection due to other organism,not elsew.class.
	0090	Infectious colitis, enteritis, & gastroenteritis
	0090	Infectious colitis, enteritis, and gastroenteritis
	0091	Colitis,enteritis,gastroenteritis of presumed inf. Origin
	0092	Infectious diarrhea
	129	Intestinal parasitism, unspecified
	00842	Intestinal infec. Due to pseudomonas

	00843	Intestinal infec. Due to campylobacter
	00845	Intestinal infec. Due to clostridium difficile
	00861	Enteritis due to rotavirus
	00862	Enteritis due to adenovirus
	00865	Enteritis due to calicivirus
	00867	Enteritis due to enterovirus, n.e.c.
	00869	Other viral enteritis
	1274	Enterobiasis
	1278	Mixed intestinal helminthiasis
	1279	Intestinal helminthiasis, unspecified
	1289	Helminth infection, unspecified
	5902	Renal and perinephric abscess
	V023	Carrier or susp.carrier of other gastrointestinal pathogens
**Central nervous system (CNS) infection**	0022	Paratyphoid fever b
	0023	Paratyphoid fever c
	048	Other enterovirus diseases of central nervous system
	320	Bacterial meningitis
	322	Meningitis of unspecified cause
	325	Phlebitis and thrombophlebitis of intracranial venous sinuses
	326	Late effects of intracranial abscess or pyogenic infection
	0470	Meningitis due to coxsackie virus
	0471	Meningitis due to echo virus
	0478	Other specified viral meningitis
	0479	Unspecified viral meningitis
	0491	Non-arthopod-borne meningitis due to adenovirus
	0499	Unsp.non-arthropod-borne viral dis.of central nervous system
	0630	Russian spring-summer (taiga) encephalitis
	3200	Hemophilus meningitis
	3201	Pneumococcal meningitis
	3202	Streptococcal meningitis
	3203	Staphylococcal meningitis
	3207	Meningitis in other bacterial diseases classified elsewhere
	3208	Meningitis due to other specified bacteria
	3209	Meningitis due to unspecified bacterium
	3229	Meningitis, unspecified
	3236	Postinfectious encephalitis
	3238	Other causes of encephalitis
	3238	Other causes of encephalitis, myelitis and encephalomyelitis
	3239	Unspecified cause of encephalitis
	3239	Unspecified cause of encephalitis, myelitis,and encephalomyelitis

	3240	Intracranial abscess
	3241	Intraspinal abscess
	3249	Intracranial and intraspinal abscess of unspecified site
	06641	West nile fever with encephalitis
	32082	Meningitis due to gram-negative
	32089	Meningitis due to other specified bacteria
	32361	Infectious acute disseminated encephalomyelitis (adem)
	32381	Other causes of encephalitis and encephlomyelitis
	32382	Other causes of myelitis
	0360	Meningococcal meningitis
**Extraintestinal salmonella infection**	0031	Salmonella septicemia
	0038	Other specified salmonella infections
	00321	Salmonella meningitis
	00323	Salmonella arthritis
	00329	Other localized salmonella infections
**H. Pylori infection**	04186	Helicobacter pylori (h. Pylori) infection
**Tuberculosis infection**	0119	Unspecified pulmonary tuberculosis
	01090	Prim. Tb. Infec., unsp.type, unsp.examination
	01120	Tb. Of lung + cavitation, unsp. Examination
	01190	Unsp. Pulmonary tb., unsp. Examination
	01194	Unsp. Pulmonary tb., found by bacterial culture
	01304	Tb. Meningitis, found by bacterial culture
	01311	Tuberculoma of meninges, bact/histol. Exam. Not done
	01394	Unsp. Tb. Of c.n.s. found by bacterial culture
	01404	Tb. Peritonitis, found by bacterial culture
	01485	Other tb. Intestines, confirmed histologically
	V1201	Personal history of tuberculosis
**Opporunistic rare infections**	022	Anthrax
	0073	Intestinal trichomoniasis
	0074	Cryptosporidiosis
	0075	Cyclosporiasis
	0082	Intestinal infec. Due to aerobacter aerogenes
	0205	Pneumonic plague, unspecified
	0209	Plague, unspecified
	0783	Cat-scratch disease
	0810	Murine (endemic) typhus
	0820	Spotted fevers
	0830	Q fever
	0838	Other specified rickettsioses
	0839	Rickettsiosis, unspecified
	00841	Intestinal infec. Due to staphylococcus
	0846	Malaria, unspecified

	0859	Leishmaniasis, unspecified
	0879	Relapsing fever, unspecified
	0909	Congenital syphilis, unspecified
	0940	Tabes dorsalis
	0980	Gonococcal infec.,acute, of lower genitourinary tract
	1000	Leptospirosis icterohemorrhagica
	1160	Blastomycosis
	1173	Aspergillosis
	1175	Cryptococcosis
	1177	Zygomycosis (phycomycosis or mucormycosis)
	1179	Other and unspecified mycoses
	1209	Schistosomiasis, unspecified
	1225	Echinococcus multilocularis infection of liver
	1228	Echinococcosis, unspecified, of liver
	1229	Echinococcosis, other and unspecified
	1270	Ascariasis
	1363	Pneumocystosis
	1369	Unsp. Infectious & parasitic diseases
	1369	Unspecified infectious and parasitic diseases
	04183	Other clostridium perfringens infection
	08881	Lyme disease (erythema chronicum migrans)
	08882	Babesiosis
	09840	Gonococcal conjunctivitis (neonatorum)
	09882	Gonococcal meningitis
	09886	Gonococcal peritonitis
**Brucellosis**	0239	Brucellosis, unspecified
	0269	Unspecified rat-bite fever
	0270	Listeriosis
	0312	Disseminated disease due to other mycobacteria
	0319	Unspecified diseases due to mycobacteria
	03283	Diphtheritic peritonitis
**Infections NOS**	0418	Other spec.bacterial inf;in condit.class.elsewhere,unsp.site
	0419	Unsp. Bacterial infection, unsp. Site
	0419	Unspec.bacterial inf;in condit. Classif.elsewhere,unsp.site
	04189	Other specified bacteria infection
	99762	Infection (chronic) of amputation stump
	V091	Infection with microorgan. Resistant to cephalosporins/b-lactam antibiotics
	V0980	Infection with microorgan. Resistant to other spec. Drugs, without mention of resistance to multiple drugs
	V0991	Infection with drug-resistant microorgan.,unspec. Drug resistance nos, with multiple drug resistance

**Pertussis**	0330	Whooping cough due to bordetella pertussis (b. Pertussis)
	0331	Whooping cough due to bordetella parapertussis
	0339	Whooping cough, unspecified organism
**Streptococcus and staphylococcus infections**	034	Streptococcal sore throat and scarlet fever
	035	Erysipelas
	0340	Streptococcal sore throat
	0341	Scarlet fever
	0410	Streptococcus infect.in condition classif.elsewhere;unsp.site
	0411	Staphylococcus infect.in condit.classif.elsewhere,unsp.site
	0411	Staphylococcus infection, unsp. Site
	0412	Pneumococcus infect.in condition classif.elsewhere;unsp.site
	0412	Pneumococcus infection, unsp. Site
	04100	Streptococcus infec., unsp.
	04101	Streptococcus infec., group a
	04101	Streptococcus infec., group a (additional code)
	04102	Streptococcus infec., group b
	04103	Streptococcus infec., group c
	04104	Streptococcus infec., group d (enterococcus)
	04105	Streptococcus infec., group g
	04109	Other streptococcus infec.
	04110	Staphylococcus infection, unsp.
	04111	Methicillin susceptible staphylococcus aureus
	04111	Staphylococcus aureus infec.
	04111	Staphylococcus aureus infec. (additional code)
	04112	Methicillin resistant staphylococcus aureus
	04119	Other staphylococcus infection
	V0252	Carrier or susp. Carrier of other streptococcus
	V0259	Carrier/susp. Carrier of other specified bacterial dis.
**Meningococcal infection**	0362	Meningococcemia
	0369	Meningococcal infection, unspecified
	03682	Meningococcal arthropathy
	03689	Other specified meningococcal infections
**Sepsis**	0380	Streptococcal septicemia
	0381	Staphylococcal septicemia
	0382	Pneumococcal septicemia
	0383	Septicemia due to anaerobes
	0388	Other specified septicemias
	0389	Unspecified septicemia
	03810	Staphylococcal speticemia, unsp.
	03811	Methicillin susceptible staphylococcus aureus septicemia
	03811	Staphylococcus aureus septicemia
	03812	Methicillin resistant staphylococcus aureus septicemia

	03819	Other staphylococcal septicemia
	03840	Septicemia due to gram-negative organism, unspecified
	03841	Septicemia due to hemophilus influenzae (h. Influenzae)
	03842	Septicemia due to escherichia coli (e. Coli)
	03843	Septicemia due to pseudomonas
	03849	Other septicemia due to gram-negative organisms
	038491	Klebsiella septicemia
	0388 1	Candida septicemia
	0388 2	Enterococcus septicemia
	0388 3	Acitinobacter septicemia
**Gram-negative infection**	0413	Friedlander's bacillus, unsp. Site
	0413	Friedlander's bacillus;condition classif.elsewhere,unsp.site
	0413	Klebsiella pneumoniae
	0414	Escherichia coli (e. Coli), unsp. Site
	0414	Escherichia coli (e. Coli), unsp. Site (additional code)
	0414	Escherichia coli(e. Coli),conditi.classif.elsewhere,unsp.site
	0415	Hemophilus influenzae in condit.classif.elsewhere,unsp.site
	0415	Hemophilus influenzae, unsp. Site (h.influenzae)
	0416	Proteus (mirabilis,morganii), unsp. Site
	0416	Proteus(mirabilis,morganii)condit,classif.elsewhere,unsp.site
	0417	Pseudomonas infec., unsp. Site
	0417	Pseudomonas infect.in condit.classif.elsewhere,unspec.site
	04185	Other gram-negative organisms infection
**Human immunodeficiency virus (HIV)**	042	Human immunodefic. Virus (hiv) dis. /aids
	V08	Asymptomatic h.i.v infection status
**Herpes, Varicella**	0521	Varicella (hemorrhagic) pneumonitis
	0527	Chickenpox with other specified complications
	0528	Chickenpox with unspecified complication
	0529	Varicella without mention of complication
	0539	Herpes zoster without mention of complication
	0540	Eczema herpeticum
	0542	Herpetic gingivostomatitis
	0543	Herpetic meningoencephalitis
	0546	Herpetic whitlow
	0549	Herpes simplex without mention of complication
	0559	Measles without mention of complication
	0569	Rubella without mention of complication
	0570	Erythema infectiosum (fifth disease)
	0578	Other specified viral exanthemata
	0579	Viral exanthem, unspecified
	0723	Mumps pancreatitis

Viral infection NOS

	0729	Mumps without mention of complication
	0740	Herpangina
	0743	Hand, foot, and mouth disease
	05311	Geniculate herpes zoster
	05319	Herpes zoster + other nervous system complications
	05320	Herpes zoster dermatitis of eyelid
	05329	Herpes zoster + other ophthalmic complications
	05410	Genital herpes, unspecified
	05440	Herpes simplex + unsp. Ophthalmic complication
	05441	Herpes simplex dermatitis of eyelid
	05443	Herpes simplex disciform keratitis
	05449	Herpes simplex with other ophthalmic complications
	05472	Herpes simplex meningitis
	05479	Herpes simplex + other spec. Complications
	05810	Roseola infantum, unspecified
	05829	Other human herpesvirus encephalitis
	0784	Foot and mouth disease
**Hepatitis**	0701	Viral hepatitis a without hepatic coma
	0701	Viral hepatitis a without mention of hepatic coma
	0709	Unsp. Viral hepatitis without hepatic coma
	0709	Unspecified viral hepatitis without mention of hepatic coma
	07030	Viral hepatitis b without hepatic coma & hepatitis delta -92
	07030	Viral hepatitis b without hepatic coma,ac/unsp.without hep. Delta
	07032	Viral hepatits b without hepatic coma,chr. Without hepatitis delt
	V0261	Hepatitis b carrier
	V0262	Hepatitis c carrier
**EBV, CMV**	075	Infectious mononucleosis
	0785	Cytomegalic inclusion disease
	0785	Cytomegaloviral disease
**Warts**	0780	Molluscum contagiosum
	0781	Viral warts
	07810	Viral warts, unspecified
	07811	Condyloma acuminatum
	07812	Plantar wart
	07819	Other specified viral warts
**Viral infection NOS**	0790	Adenovirus inf.in conditions classif.elsewhere,unsp.site
	0790	Adenovirus infection, unsp. Site
	0791	Echo virus infection, unsp. Site
	0792	Coxsackie virus infection, unsp. Site
	0793	Rhinovirus infection, unsp. Site

	0798	Other specified viral infection classif.elsewhere,unsp.site
	0799	Unsp. Viral & chlanydial infection
	0799	Unsp.viral infect.in conditions classif.elsewhere,unsp.site
	07889	Other specified diseases due to viruses
	07889	Other specified diseases due to viruses and chlamydiae
	07950	Retrovirus, unsp.,unsp. Site
	07959	Other specified retrovirus
	07989	Other spec. Viral infection
	07999	Unsp. Viral infection
	07999	Unsp. Viral infection (additional code)
**RSV infection**	0796	Respiratory syncytial virus (rsv)
**Fungal infection**	1100	Dermatophytosis of scalp and beard
	1101	Dermatophytosis of nail
	1103	Dermatophytosis of groin and perianal area
	1104	Dermatophytosis of foot
	1105	Dermatophytosis of the body
	1109	Dermatophytosis of unspecified site
	1110	Pityriasis versicolor
	1118	Other specified dermatomycoses
	1119	Dermatomycosis, unspecified
	1120	Candidiasis of mouth
	1121	Candidiasis of vulva and vagina
	1122	Candidiasis of other urogenital sites
	1123	Candidiasis of skin and nails
	1124	Candidiasis of lung
	1129	Candidiasis of unspecified site
	11281	Candidal endocarditis
	11282	Candidal otitis externa
	11284	Candidal esophagitis
	11289	Other candidiasis of other specified sites
**Otitis**	382	Suppurative and unspecified otitis media
	3813	Other and unspecified chronic nonsuppurative otitis media
	3814	Nonsuppurative otitis media, not specified as acute or chronic
	3819	Unspecified eustachian tube disorder
	3820	Acute suppurative otitis media
	3821	Chronic tubotympanic suppurative otitis media
	3823	Unspecified chronic suppurative otitis media
	3824	Unspecified suppurative otitis media
	3829	Unspecified otitis media
	3831	Chronic mastoiditis
	3839	Unspecified mastoiditis

	3841	Chronic myringitis without mention of otitis media
	3849	Unspecified disorder of tympanic membrane
	38001	Acute perichondritis of pinna
	38002	Chronic perichondritis of pinna
	38003	Chondritis of pinna
	38010	Infective otitis externa, unspecified
	38015	Chronic mycotic otitis externa
	38022	Other acute otitis externa
	38023	Other chronic otitis externa
	38100	Acute nonsuppurative otitis media, unspecified
	38101	Acute serous otitis media
	38104	Acute allergic serous otitis media
	38110	Chronic serous otitis media, simple or unspecified
	38119	Other chronic serous otitis media
	38129	Other chronic mucoid otitis media
	38200	Ac.suppurat.otitis media without spontan.rupture of eardrum
	38201	Acute suppurat.otitis media with spontan.rupture of eardrum
	38300	Acute mastoiditis without complications
	38301	Subperiosteal abscess of mastoid
	38330	Postmastoidectomy complication, unspecified
	38389	Other disorders of mastoid
	38400	Acute myringitis, unspecified
	38401	Bullous myringitis
	38420	Perforation of tympanic membrane, unspecified
	38482	Atrophic nonflaccid tympanic membrane
	38500	Tympanosclerosis, unspecified as to involvement
	38510	Adhesive middle ear disease, unspecified as to involvement
	38530	Cholesteatoma, unspecified
	38531	Cholesteatoma of attic
	38532	Cholesteatoma of middle ear
	38630	Labyrinthitis, unspecified
	38635	Viral labyrinthitis
**Upper respiratory infection**	460	Acute nasopharyngitis (common cold)
	462	Acute pharyngitis
	463	Acute tonsillitis
	464	Acute laryngitis and tracheitis
	465	Acute upper respiratory infections of multiple or unsp.sites
	475	Peritonsillar abscess
	4610	Acute maxillary sinusitis
	4611	Acute frontal sinusitis
	4612	Acute ethmoidal sinusitis

	4613	Acute sphenoidal sinusitis
	4618	Other acute sinusitis
	4619	Acute sinusitis, unspecified
	4640	Acute laryngitis
	4644	Croup
	4650	Acute laryngopharyngitis
	4658	Acute upper respiratory infections of other multiple sites
	4659	Acute upper respiratory infections of unspecified site
	4720	Chronic rhinitis
	4730	Chronic maxillary sinusitis
	4731	Chronic frontal sinusitis
	4732	Chronic ethmoidal sinusitis
	4733	Chronic sphenoidal sinusitis
	4738	Other chronic sinusitis
	4739	Unspecified sinusitis (chronic)
	4741	Hypertrophy of tonsils and adenoids
	4742	Adenoid vegetations
	4748	Other chronic disease of tonsils and adenoids
	4749	Unspecified chronic disease of tonsils and adenoids
	4870	Influenza with pneumonia
	4871	Influenza with other respiratory manifestations
	4878	Influenza with other manifestations
	4880	Influenza due to identified avian influenza virus
	4881	Influenza due to identified novel h1n1 influenza virus
	46400	Acute laryngitis without mention of obstruction
	46410	Acute tracheitis without mention of obstruction
	46420	Acute laryngotracheitis without mention of obstruction
	46430	Acute epiglottitis without mention of obstruction
	46450	Supraglottitis without mention of obstruction
	47400	Chronic tonsillitis
	47401	Chronic adenoiditis
	47410	Hypertrophy of tonsil with adenoids
	47411	Hypertrophy of tonsils alone
	47412	Hypertrophy of adenoids alone
	47822	Parapharyngeal abscess
	47824	Retropharyngeal abscess
**Bronchiolitis**	466	Acute bronchitis and bronchiolitis
	480	Viral pneumonia
	490	Bronchitis, not specified as acute or chronic
	4660	Acute bronchitis
	4661	Acute bronchiolitis
	4800	Pneumonia due to adenovirus

	4801	Pneumonia due to respiratory syncytial virus
	4802	Pneumonia due to parainfluenza virus
	4808	Pneumonia due to other virus not elsewhere classified
	4809	Viral pneumonia, unspecified
	4841	Pneumonia in cytomegalic inclusion disease
	4918	Other chronic bronchitis
	46611	Ac. Bronchiolitis due to respiratory syncytial virus (rsv)
	46619	Ac. Bronchiolitis due to other infectious organisms
	49121	Obstructive chr. Bronchitis with(acute)exacerbation
	V1261	Personal history of pneumonia (recurrent)
**Pneumonia**	481	Pneumococcal pneumonia
	481	Pneumococcal pneumonia (streptococuus pneumoniae pneumonia)
	483	Pneumonia due to other specified organism
	4820	Pneumonia due to klebsiella pneumoniae
	4821	Pneumonia due to pseudomonas
	4822	Pneumonia due to hemophilus influenzae (h. Influenzae)
	4823	Pneumonia due to streptococcus
	4829	Bacterial pneumonia, unspecified
	4830	Pneumonia due to mycoplasma pneumoniae
	4831	Pneumonia deu to chlamydia
	4838	Pneumonia due to other specified organism
	48230	Pneumonia due to streptococcus, unspecified
	48231	Pneumonia due to streptococcus, group a
	48241	Pneumonia due to staphylococcus aureus
	99590	Systemic inflammatory response syndrome, unspecified
	99591	Sepsis
	99591	Systemic inflammatory response syndrome due to infectious process without organ dysfunction
	99592	Severe sepsis
	99592	Systemic inflammatory response syndrome due to infectious process with organ dysfunction
**Mycoplasma infection**	04181	Other mycoplasma infection
	04181	Other mycoplasma infection (additional code)
**Other**	101	Vincent's angina
	0400	Gas gangrene
	0990	Chancroid
	0993	Reiter's disease
	0999	Venereal disease, unspecified
	1236	Hymenolepiasis
	1307	Toxoplasmosis of other specified sites
	1309	Toxoplasmosis, unspecified

	1320	Pediculus capitis (head louse)
	1322	Phthirus pubis (pubic louse)
	1323	Mixed pediculosis infestation
	1329	Pediculosis, unspecified
	1330	Scabies
	1340	Myiasis
	04082	Toxic shock syndrome
	04089	Other specified bacterial diseases
	04184	Other anaerobes infection
	05442	Dendritic keratitis
	07988	Other spec. Chlamydial infection
	07998	Unsp. Chlamydial infection
